# Effect of Regulating Corn Glutelin Peptides via the Plastein Reaction on Its Physicochemical Characteristics and the Quality of Baked Bread

**DOI:** 10.3390/foods15071173

**Published:** 2026-03-31

**Authors:** Yang Sun, Wan-Ying Zhang, Yue-Yuan Lu, Hai-Bo Lu, Guo-Jun Du, Yue Zhao, Yonghui Sun, Li-Ying Bo, Jian Ren, Jing-Jing An, Meng Wang

**Affiliations:** 1 Faculty of Food Quality and Safety, Qiqihar University, Qiqihar 161006, China; 2 Tianjin Institute of Industrial Biotechnology, Chinese Academy of Sciences, Tianjin 300308, China; 3 Haihe Laboratory of Synthetic Biology, 21 West 15th Avenue, Tianjin Airport Economic Area, Tianjin 300308, China

**Keywords:** corn glutelin hydrolysate, plastein reaction, functional properties, structural characteristics, baked goods

## Abstract

Corn glutelin is the main protein component of corn processing by-products, with a wide range of sources and low cost. However, its hydrophobic molecular structure, poor solubility, foaming and emulsifying properties limit its application in the food industry. Enzymatic hydrolysis can effectively improve its solubility, but the functional properties of hydrolysis products still need further improvement. The plastein reaction is a mild enzymatic modification method that can recondense small peptides in hydrolysis products under the catalysis of protease, meanwhile introducing exogenous amino acids to achieve the targeted regulation of product structure and function. Corn glutelin was hydrolyzed to obtain corn glutelin hydrolysate (CGH). Corn glutelin hydrolysate (CGH) with exogenous amino acids (valine, tyrosine, cysteine and threonine) was mediated by plastein reaction in order to gain modified products enriched with these amino acids, which are Val-CGH, Tyr-CGH, Cys-CGH and Thr-CGH, respectively. This study mainly investigated the functional properties and structural characteristics of these modified peptides. Simultaneously, the modified peptides with superior solubility, foaming ability and foaming stability were screened and applied to bread formulas to evaluate potential application of plastein reaction modifiers in the baking field. The effects of modified peptides on the specific volume of dough, texture and sensory properties of bread were assessed. Among the modified peptides, Cys-CGH had the best foaming property and foaming stability, and fine solubility. Compared with CGH, the solubility of Cys-CGH increased by 4.16%, foaming performance (FC) increased by 41.5%, foaming stability at 10 min (FS10) increased by 10.44%, foaming stability at 20 min (FS20) improved by 12.67%, and bubble stability at 30 min (FS30) improved by 16.63%. In addition, the baking loss rate of the bread sample containing 0.5% Cys-CGH decreased by 0.93%, the specific volume enhanced by 0.27 cm^3^/g, the hardness lowered by 0.3 N, the springiness raised by 1.03, the chewiness improved by 7.5 N. The sensory acceptance of bread samples with 0.5% Cys-CGH was significantly optimized. In brief, this also demonstrates that adding modifiers with good functional properties can improve the quality of baked products, highlighting their potential as a green food additive in baked goods.

## 1. Introduction

In modern society, the use of food additives has become an integral part of the food industry and human life needs, which plays a key role in endowing food with a superior taste, extending shelf life, and strengthening color and stability. However, with the continuous improvement of public health awareness, the safety of food additives has attracted increasing attention, especially its potential health hazards, which has gradually become the focus of scientific research and national supervision. For example, sodium benzoate may cause a series of adverse health reactions, one of the most common health hazards being its potential to induce or aggravate allergic reactions, including asthma, urticaria and skin itching [1]. In contrast, green food additives based on natural ingredients are gradually promoting the development of the industry in a more sustainable direction because of their advantages of high safety and environmental friendliness. Studies have demonstrated that shortening, as a common baking ingredient, can lead to higher levels of saturated and trans fatty acids. Regular fat intake is bound up with multiple adverse reactions on health, including abnormal blood lipids and cardiovascular problems. Kim et al. used oleogels instead of shortening to produce white bread. During the white bread processing, replacing shortening with fat gel can reduce the content of saturated fat and trans fat. When fat gel completely replaced shortening, the saturated fatty acids reduced by 52.46%, and the trans-fatty acids reduced by 75.72%. Antioxidants, as food additives, are used to prevent the oxidative deterioration of oils and fats in processed foods [2]. However, some synthetic antioxidants exhibit toxicity, mutagenicity and carcinogenicity [3]. Correspondingly, Lu et al. studied the substitution of antioxidants with different proportions of green tea powder (10%, 20%, and 30%) during cake preparation [4]. The results indicated that compared to the cake sample without green tea powder, the specific volume of the cake sample with 30% green tea powder increased by 0.132 g/cm^3^, the chewiness was enhanced by 19.1 g, and its antioxidant activity also improved. As an important natural food, cereals are abundant in dietary fiber, vitamins, minerals and phytochemicals, making them ideal carriers for food ingredients and functional foods. In addition, black rice is high in anthocyanins, vitamin E, γ-oryzanol and a variety of minerals, which collectively endow it with powerful antioxidant, anti-inflammatory and cardiovascular protection functions [5,6]. Meanwhile, both free and bound phenolic extracts derived from wheat showed strong antioxidant activity [7]. Accordingly, cereals and their corresponding products have attracted more and more attention in the food industry due to various bioactive components.

Maize is one of the leading cereal crops worldwide. Maize plays an indispensable role in the fields of food, feed and industrial raw materials. Corn gluten meal (CGM) is the main byproduct during the production of corn starch by wet milling, which is rich in protein resource [8]. The proteins in corn gluten meal primarily consist of 65–68% zein, 22–33% glutelin, and small amount of globulin and albumin [9,10]. Glutelin has a large content of hydrophobic amino acid residues and forms a stable aggregation structure through disulfide bonds, which makes it difficult to disperse in aqueous systems [9]. Therefore, corn glutelin has poor solubility, only soluble in alkali and dilute acid reagents, which greatly restricts its utilization in the food field. Hydrolysis serves as an efficient strategy for improving the functional performance of proteins. However, bioactive peptides exhibit insufficient stability, and their functional activity and thermal stability are affected by environmental factors. Therefore, enhancing the stability of bioactive peptides and exploring their new applications in the food industry have been a long-term focus in the scientific research field. Studies have confirmed that compared with whey protein isolate (WPI), the conjugate of WPI and epigallocatechin gallate (EGCG), WPI-EGCG, enhanced the radical scavenging activity against DPPH and ABTS by 42.26% and 37.99%, respectively [11]. The covalent binding of rice protein hydrolysate (RPH) and chlorogenic acid (CA) can enhance its emulsifying activity. The emulsifying parameter of rice protein hydrolysate was 22.51 m^2^/g, but it significantly increased to 25.41 m^2^/g after covalent binding [12]. Additionally, studies have verified that compared with soy protein concentrate hydrolysate (SCPH), the emulsion stability of SCPH modified by the plastein reaction increased by threefold [13]. Chicken lung protein hydrolysate (CLPH) by the plastein reaction was modified, which could improve the foaming and emulsifying properties of CLPH, and the DPPH free radical scavenging activity (from 30.04% to 71.74%) and ABTS free radical scavenging activity (from 41.66% to 62.44%) [14]. The plastein reaction could also enhance the surface hydrophobicity and bile acid binding capacity of chicken protein hydrolysates [15]. The plastein reaction may reshape the amino acid sequence as well as the three-dimensional structure of polypeptides and proteins, effectively modifying bioactive peptides and improving the functional properties and nutritional value of proteins [16]. More importantly, the reagents involved in the plastein reaction are all food-grade. Meanwhile, earlier studies have already confirmed that surface hydrophobicity and solubility are the key factors affecting the foaming characteristics of proteins [17]. Thus, the plastein reaction can increase the surface hydrophobicity of protein, improve the adsorption ability of protein on the gas–liquid interface, and then enhance the stability and formation ability of foam.

The sulfhydryl group in the cysteine molecule can act as a nucleophile to perform an addition reaction with acrylamide, converting it into a non-toxic derivative. Cysteine can efficiently and quantitatively eliminate acrylamide under heating conditions below 120 °C [18,19]. Cysteine excels in inhibiting food browning, and research has shown that adding 0.1% cysteine is effective in preventing internal browning of Stanley plums during refrigeration [20]. Valine is one of the key amino acids to maintain protein synthesis and nitrogen balance. For animals or fish at a particular growth stage, the concentration of valine in the diet directly affects their growth performance. For example, in a study on juvenile red-fin puffer (*Pagrus major*), it was found that feed with a valine addition level of ≥0.79% markedly improved the proliferation and feed utilization efficiency of fish fed diets, compared to feed with a valine addition level of 0.27% (*p* < 0.05). When the valine level exceeded 0.79%, the water content in the fish body obviously reduced, and the protein content increased [21]. The phenolic hydroxyl, α-amino and α-carboxyl groups present in tyrosine confer high reactivity, which not only makes it the basic unit of protein synthesis, but also plays a critical impact on key processes such as flavor formation, nutrition enhancement, and enzymatic crosslinking [22]. Threonine participates in Maillard reaction and influences the formation of color and flavor compounds. Specific lactic acid bacteria such as *Lactobacillus delbrueckii* subsp. Bulgaria is capable of efficiently utilizing threonine during dough fermentation and converting it into key aromatic compounds, such as acetaldehyde, through the action of threonine deaminase [23]. Thus, it endows fermented food with unique fresh aroma and flavor characteristics.

At present, investigations into corn-derived peptides mainly concentrate on high F-ratio oligopeptides, antihypertensive peptides, and anti-hangover peptides. However, there is relatively limited research on optimizing the functional properties of corn glutelin hydrolysates through the plastein reaction and their application in the baking industry. In the current study, corn glutelin was treated with alkaline protease for hydrolysis to obtain target peptides, which were further modified via the plastein reaction in the presence of exogenous amino acids (valine, tyrosine, cysteine and threonine). The structural and functional characteristics of the modified peptides were evaluated; the modified peptides with superior solubility, foaming property and foaming stability were selected. Hence, the impact of corn glutelin hydrolysate refined by means of the plastein reaction on bread quality was estimated. The objective of this study was to refine the physical and chemical properties of corn glutelin hydrolysates to enhance the bioavailability of corn glutelin and to develop novel applications in the baking sector.

## 2. Materials and Methods

### 2.1. Materials and Reagents

Corn gluten meal was purchased from Heilongjiang Beidahuang Group Co., Ltd. (Harbin, China). L-valine (Val) and L-threonine (Thr) were obtained from Solarbio Science and Technology Co., Ltd. (Beijing, China). L-tyrosine (Tyr) and L-cysteine (Cys) were derived from Macklin Biochemical Technology Co., Ltd. (Shanghai, China). Flour used for bread sample preparation was purchased from Whole Grain Food Co., Ltd. (Xinxiang, Henan, China). Alkaline proteolytic enzyme (enzyme activity: 20,000 U/g) was obtained from Solarbio Science and Technology Co., Ltd. (Beijing, China). Whole milk powder was derived from Beidahuang Wandashan Dairy Co., Ltd. (Harbin, China). Butter was sourced from Fonterra Trading Co., Ltd. (Shanghai, China). All other chemicals employed in the present research were of analytical purity and bought from commercial sources. All other chemicals used in this research were of analytical purity and bought from commercial suppliers.

### 2.2. Corn Glutelin Extraction and Its Hydrolysates Preparation

The extraction of corn glutelin and preparation of its hydrolysates were performed according to the minor method of Sun et al. [24]. Corn glutelin (CG) and corn glutelin hydrolysate (CGH) were obtained. A PBS (Phosphate-buffered solution) solution (20 mmol/L) with the pH value of 6.5 was prepared. Corn gluten meal was mixed with PBS (Phosphate-buffered solution) in a ratio of 1:10 (*w*/*v*), then 1% (*w*/*v*) α-amylase (3700 U/g) was added. The solution was hydrolyzed at 70 °C for 2 h; the pH value was measured every 30 min during this process. During the reaction, NaOH solution (1 mol/L) was employed to adjust the pH so as to maintain it within the range of 5.5–7.5. Then, the enzyme was inactivated for 15 min in a boiling water bath, followed by cooling and centrifugation for 10 min at 4000 r/min. The resulting precipitate was washed three times with deionized water, dried, and ground into powder for later use. The above powder was dissolved in absolute ethanol (*w*/*v*, 1:10), stirred for 2 h, then centrifuged at 4000 r/min for 10 min. The obtained powder was extracted with 0.1 mol/L NaOH solution at 60 °C for 2 h, then centrifuged at 4000 r/min for 20 min. The pH of the supernatant was adjusted to 4.8 using 4 mol/L HCl and the supernatant was centrifuged again at the same speed and time as above. The obtained precipitate was washed three times using 70% ethanol, washed with distilled water with pH of 4.8 twice, and then dissolved in a small amount of water. Its pH was adjusted to 8.0 using 2 mol/L NaOH, and the resulting solution was freeze-dried for 48 h to obtain corn glutelin (CG).

Corn glutelin was mixed with pH 8 distilled water at a ratio of 1:20 (*w*/*v*) and then hydrolyzed with 3% alkaline protease (2.0 × 10^5^ U/g) at 50 °C for 8 h. During the entire reaction, the pH was kept constant at 8.0 using NaOH solution (1 mol/L). After the reaction, the mixture was kept in boiling water for inactivation for 15 min, followed by centrifugation at 4000 r/min for 30 min. The supernatant was freeze-dried to yield corn glutelin hydrolysate (CGH). The supernatant was lyophilized to obtain and calculate the corn glutelin hydrolysate (CGH) with a protein content of 84.7%.

### 2.3. Preparation of Plastein Reaction Modifiers

The plastein reaction was performed with 1% alkaline protease (2.0 kU/g) and 40% CGH (*w*/*w*) at 45 °C for 7 h, and the addition ratios of exogenous amino acids (Val, Tyr, Cys and Thr) were 0.7 mol/mol free amino acids. The reaction mixture was heated in a water bath at 95 °C for 15 min to inactivate the enzyme at reaction termination. The obtained modifiers (Val-CGH, Tyr-CGH, Cys-CGH, and Thr-CGH) were lyophilized and stored for future experimental use.

### 2.4. Measurement of Free Amino Amount and Hydrolysis Degree

The free amino acid level and hydrolysis degree of these samples were detected according to the OPA method [25], with slight modifications. The OPA reagent was prepared by dissolving 1.9068 g Na_2_B_4_O_7_ (sodium tetraborate), 0.088 g DTT (dithiothreitol), and 0.1 g SDS (sodium dodecyl sulfate) in water, and then 0.080 g OPA was solubilized in the dark in absolute ethanol (2 mL). All solutions were then volumed in a 100 mL brown volumetric flask. In total, 3 mL of sample solution was mixed with 3 mL of OPA reagent. After reaction in the dark (5 min), the absorbance at 340 nm was detected with a UV-Vis spectrophotometer (TU-1810, Beijing Puxi General Instrument Co., Ltd., Beijing, China).

$$Y=\frac{A\;\times \;N}{131.17\;\times \;X}$$

Y: Free amino amount (mmol/g); *X*: Protein mass per unit volume (g/L); *N*: Dilution factor; *A*: Leucine content (µg/mL); 131.17: Molecular weight of leucine (g/mol);

$$\mathrm{DH}\;\left(\%\right)=\frac{Y_{2}\;-\;Y_{1}}{h_{\mathrm{tot}}}\;\times \;1$$

Y_1_: Level of free amino groups before hydrolysis (mmol/L); Y_2_: The level of free amino acid in the hydrolyzed protein (mmol/L); h_tot_: Gram equivalent weight of peptide bond per gram of protein (Corn Protein 8.38).

### 2.5. Detection of Physicochemical Properties of These Modifiers

#### 2.5.1. Solubility Detection

The Folin phenol method was applied to measure the soluble protein concentration, based on the method provided by Xue et al. [26], with minor modifications. Deionized water with different pH concentrations (2.0, 3.0, 4.0, 5.0, 6.0, 7.0, 8.0, 9.0, and 10.0) was prepared with 0.1 mol/L NaOH solution and 0.1 mol/L HCL solution. The 5 mg/mL sample solution was prepared using deionized water with different pH levels. The absorbance value of the collected supernatant was determined.

$$Solubility\left(\%\right)=\frac{\mathrm{Supernatant}\;\mathrm{protein}\;\mathrm{content}}{Total\;protein\;content}\times 100$$

#### 2.5.2. Detection of Emulsification Activity (EAI) and Emulsion Stability (ESI)

Emulsification and emulsifying stability were detected by the method derived from Wang et al. [27], with tiny alterations. A sample solution (2 mg/mL) was prepared and mixed with soybean oil at a volume ratio of 3:1. The mixture was dispersed with a high shear machine for 2 min at the speed of 14,000 r/min. At 0 min and 30 min, 50 μL sample was taken out from the bottom of the centrifuge tube and diluted 100 times using 0.1% SDS solution. The resulting solution absorbance value was measured at 500 nm. The SDS solution served as a blank. The formulas of EAI and ESI are as follows:

$$\mathrm{EAI}\;=\;\frac{2\;\times \;2.303\;\times \;A_{0}\;\times \;\mathrm{DF}}{C\;\times \;\varphi \;\times \;\theta \;\times \;10,000}$$

$$\mathrm{ESI}=\frac{A_{0}\;\times \;30}{A_{0}\;-\;A_{30}}$$

A_0_: Absorbance value of emulsion volume at 0 min; DF: Dilution factor, 100; φ: Optical path, 1 cm; C: Protein content, g/mL; θ: Oil volume fraction, 0.25; A_30_: Absorbance value of emulsion volume at 30 min.

#### 2.5.3. Detection of Foaming Ability and Foaming Stability

The determination of foaming ability and foaming stability, based on the method provided by Melchior et al. [28] with minor changes. A 1% protein concentration solution was prepared and placed in a 50 mL centrifuge tube and homogenized for 2 min at 10,000 rpm. The foam height was directly recorded using the centrifuge tube scale. The foam height after homogenization is expressed as H_1_. After standing for 10 min, 20 min and 30 min, the foam heights were recorded as H_2_, H_3_ and H_4_, respectively. The formulas are as follows:

$$\mathrm{FA}(\%)\;=\;\frac{H_{1}}{H_{0}}\;\times \;100\%$$

$$\mathrm{FS}(\%)=\frac{H_{2}}{H_{1}}\;\times \;100\%$$

H_0_: Initial height (cm); H_1_: after homogenization, the initial foam height (cm); H_2_: Foam height at 10 min (cm); H_3_: Foam height at 20 min (cm); H_4_: Foam height at 30 min (cm).

### 2.6. Detection of Particle Size and Zeta Potential

A 1 mg/mL sample solution was prepared and its Zeta potential was detected using a Zeta potential analyzer (ZS90, Malvern Panalytical Ltd., Worcestershire, UK) according to the method used by Zhou et al. [13].

### 2.7. Scanning Electron Microscopy (SEM) Observation of the Microstructure of the Modifier Samples

Adopt the method published by Zhou et al. [29] with minor modifications. The microstructures of CGH, Cys+CGH and Cys-CGH were analyzed. The bread powder sample using a toothpick was fixed on glass slides with double-sided adhesive tape for gold vacuum spraying. The sample was observed and photographed at 10 kV with a scanning electron microscope (SEM, model SU3500, Hitachi, Tokyo, Japan).

### 2.8. Detection of Amino Acid Composition

Amino acid content was detected referring to the method reported by Zheng et al. [9], with slight modifications. The amino acid composition was measured with Hitachi amino acid analyzer LA8080 (Hitachi, Japan) by hydrolyzing the samples according to the GB 5009.124-2016 [30]. The sample was mixed with 6 M HCl, placed in a hydrolysis tube, and 3 drops of phenol were added. It was then filled with nitrogen and sealed, hydrolyzed at 110 °C for 22 h, and then the hydrolysate was filtered and volumed in a 50 mL volumetric flask. In total, 1 mL filtrate was shifted to test tube and then dried under reduced pressure. Then it was dissolved in sodium citrate buffer solution (2 mL, pH 2.2) and filtered using a 0.22 μm filter membrane to obtain the sample solution.

### 2.9. Analysis of the Quality of Bread Sample After Adding Modifiers

#### 2.9.1. The Preparation of Bread Samples

250 g high-gluten wheat flour, 15 g skim milk powder, 5 g yeast, 25 g egg and 130 g water were poured into a dough mixer, and 0.5%, 1% and 2% modifiers were added, respectively, and then stirred for 10 min at medium speed. In total, 35 g of butter was added and continued to mix for 15 min at medium speed. After stirring, the first fermentation is carried out (25 °C, 85%), tumbled and relaxed, shaped, and then placed into a mold, proceeding to the second fermentation (37 °C, 85%). Then, it is placed into an oven (upper heat 150 °C, lower heat 160 °C) and baked for 40 min.

#### 2.9.2. Detection of the Baking Characteristics of Bread Samples

The fermented dough and the baked bread were both weighed by an electronic balance. Bread volume was detected on the base of the rapeseed displacement method; subsequently, the specific volume was computed [31]. The calculation of baking loss was referring to the mass difference between dough weight and its weight after baking [32].

$$\mathrm{SV}=\frac{V}{M}$$

$$\mathrm{BL}(\%)=\frac{M_{1}-M}{M_{1}}\;\times \;100\%$$

SV: bread specific volume (cm^3^/g); BL: baking loss rate (%); V: bread volume (cm^3^); M: bread mass (g); M_1_: dough mass (g);

#### 2.9.3. Detection of Internal Structure of the Bread Crumb

Based on the method derived from Ma et al. [33] with slight modifications, slices of 10 cm^2^ were taken from the center of different bread samples. High-resolution picture was photographed with a high-pixel camera and converted to 8-bit. The Ostu algorithm in the ImageJ software (version v1.54p) was used to perform automatic threshold segmentation, in order to acquire the following parameter: porosity.

#### 2.9.4. Detection of Texture Characteristics of Bread Sample

Refer to the method provided by Franco et al. [34], with slight modifications. The baked bread was cut into uniform thin pieces (thickness, 1.5 cm). Determination of texture was performed with a texture analyzer (QTS-25; AMETEK Brookfield, Middleboro, MA, USA). The TPA (texture profile analysis) test parameters are as follows: the speed is 1 mm/s, the trigger force is 0.10 N, the interval between two compressions is 2 s, and the testing height is 60 mm. The hardness, springiness, adhesiveness, chewiness and cohesiveness of the bread sample were investigated.

#### 2.9.5. Detection of Bread Sample Color

According to the method of Tomić et al. [35], the three major color indexes of both the bread crust and the bread inner core of the cut slice were determined with a portable colorimeter (LS173; Shenzhen Linshang Technology Co., Ltd., Shenzhen, China). Results were represented as three coordinates in Lab color space: a from green (−) to red (+), and b from blue (−) to yellow (+), L (lightness) from black (0) to white (100).

#### 2.9.6. Electronic Tongue Determination

Electronic tongue for food is an analytical method that objectively evaluates the taste of food by mimicking human taste perception, combining chemical sensor arrays with pattern recognition technology [36]. Sourness, bitterness, astringency, aftertaste bitterness, aftertaste astringency, saltiness, umami and richness of bread samples were measured using an electronic tongue (SA402B Controller, Intelligent Sensor Co., Ltd., Atsugi, Japan).

### 2.10. Statistical Analysis of Data

All experimental indexes were independently detected in triplicate (n = 3), and all data were exhibited as mean ± standard deviation (SD). Image analysis and statistical processing were carried out referring to GraphPad Prism 8.0.2 and Excel 2011. One-way analysis of variance (ANOVA) was assessed according to Duncan’s multiple range test with significance differences (*p* < 0.05).

## 3. Results and Discussion

### 3.1. Result Analysis of Free Amino Group Content and DH of the Modifiers

The free amino levels of different samples are shown in Table 1. After hydrolysis, the free amino group concentration of the resulting CGH was 1.7928 ± 0.0170 mmol/g protein, compared to CG itself, which markedly improved by 0.0231 ± 0.0013 mmol/g protein (*p* < 0.05); meanwhile, the resultant hydrolysis degree (DH) amounted to 21.12%. Compared with CGH, the free amino level of the plastein reaction modifiers significantly decreased (*p* < 0.05). Compared with the physical mixtures (Val+CGH, Thr+CGH, Tyr+CGH and Cys+CGH), the free amino group level of these modifiers (Val-CGH, Thr-CGH, Tyr-CGH and Cys-CGH) significantly decreased by 0.4778, 0.4456, 0.2286 and 0.4366 mmol/g protein, respectively (*p* < 0.05). Plastein reaction is a peptide condensation process catalyzed by protease, with the key feature being the reassembly of small peptides and free amino acids into high molecular weight peptide polymers through condensation reaction [25]. According to the research report of Bo et al. [37], the plastein reaction can reduce the level of free amino acids. The -NH_2_ levels of CGH-Phe and CGH-Trp decreased by 0.388 and 0.394 mmol/g protein, compared to those of simple physical mixtures (CGH+Phe and CGH+Trp), respectively. It is worth mentioning that the results are similar to those declared by Shi et al. [38]. Compared with unmodified CGH+Gly, CGH+Pro and CGH+Hyp, the free amino content of CGH-Gly, CGH-Pro and CGH-Hyp decreased significantly by 0.359 mmol/g protein, 0.356 mmol/g protein and 0.364 mmol/g protein, respectively.

### 3.2. Solubility Changes in Different Modifiers

**Figure 1:**
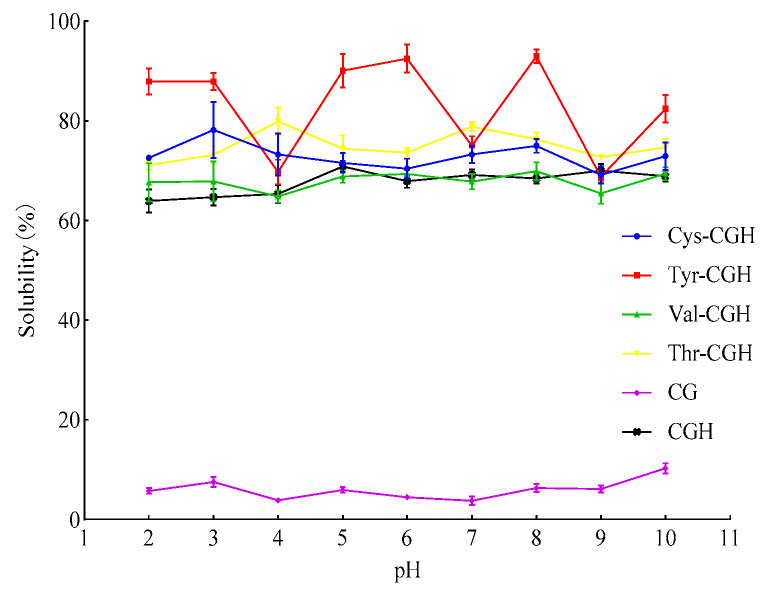
The changes in solubility of modified products at different pH values. Note: One-way ANOVA was carried out. Cys-CGH: cysteine modifier; Tyr-CGH: tyrosine modifier; Val-CGH: valine modifier; Thr-CGH: threonine modifier; CG: corn glutelin; CGH: corn glutelin hydrolysate.

The solubility features of different samples within the pH of 2–10 are presented in Figure 1. As shown in the figure, CGH-Cys showed the highest solubility at pH 8 (75.01%), CGH-Tyr had the highest solubility at pH 8 (87.12%), CGH-Val showed the highest solubility at pH 8 (69.92%) and CGH-Thr at pH 4 (79.91%). CGH showed the highest solubility (70.85%) at pH 5. The solubility of Val-CGH is slightly lower than that of CGH. The solubility of CGH-Tyr, CGH-Cys and CGH-Thr was higher than that of CG, CGH within the entire pH range investigated. The plastein reaction is essentially a retrograde condensation process catalyzed by protease under the condition of high concentration of peptide fragments, which can promote the re-polymerization of short peptides to form higher molecular weight aggregates or cross-linked networks [14]. As a hydrophobic amino acid, the hydrophobic side chain of valine can increase the hydrophobicity of protein surface, reduce the interactions with water molecules, and thus reduce the solubility of protein in aqueous solution [39]. Threonine is an important protein-derived α-amino acid. Its side chain contains a hydroxyl group (-OH), which interacts with water molecules or other polar groups through hydrogen bonds, significantly enhancing the solubility and stability of polypeptide chains in and aqueous environment [40]. Phenolic hydroxyl, as the core functional group of tyrosine side chains, can form a hydrogen bonding network with the surrounding water molecules or polar residues in the protein, thereby improving the hydrophilicity [41]. The sulfhydryl group of cysteine can also participate in the reduction reaction of the disulfide bond, which breaks down the disulfide bond into two sulfhydryl groups, increasing its exposed hydrophilic surface, and thus improving the solubility of proteins [42].

### 3.3. Changes in Emulsifying Activity and Emulsification Stability (ESI) of Different Products

**Figure 2:**
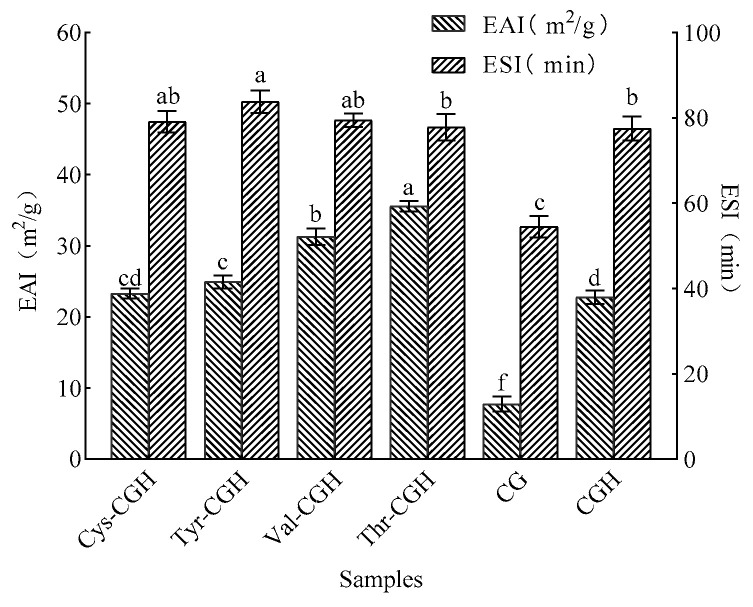
The emulsifying activity changes in different samples. Note: One-way ANOVA was carried out. Different letters in above figure demonstrate obvious differences among groups (*p* < 0.05). Cys-CGH: cysteine modifier; Tyr-CGH: tyrosine modifier; Val-CGH: valine modifier; Thr-CGH: threonine modifier; CG: corn glutelin; CGH: corn glutelin hydrolysate.

Figure 2 illustrates the emulsifying property and emulsion stability of modified products. The emulsifying activity (EAI) of Thr-CGH (35.558 m^2^/g) was significantly higher than that of Val-CGH (31.290 m^2^/g), Thr-CGH (24.934 m^2^/g), Cys-CGH (23.306 m^2^/g) and CGH (22.754 m^2^/g) (*p* < 0.05). The ESI of Cys-CGH, Tyr-CGH and Val-CGH were significantly higher than CGH (*p* < 0.05), increased by 1.59 min, 6.24 min and 1.90 min, respectively. The emulsifying property and emulsifying stability of the modifiers obviously increased, which may be due to the rearrangement and aggregation of peptide chains during the plastein reaction processing, resulting in the increased exposure of hydrophobic amino acid residues, thus enhancing the interfacial adsorption capacity [13]. Research provided by Cheng et al. indicated that the plastein reaction significantly enhanced the emulsifying capacity and emulsion stability of chicken lung protein hydrolysate (CLPH), with increases of 7.22 m^2^/g and 6.11 min, respectively [14].

### 3.4. Changes in Foam Capacity (FC) and Foam Stability (FS) of Different Products

**Figure 3:**
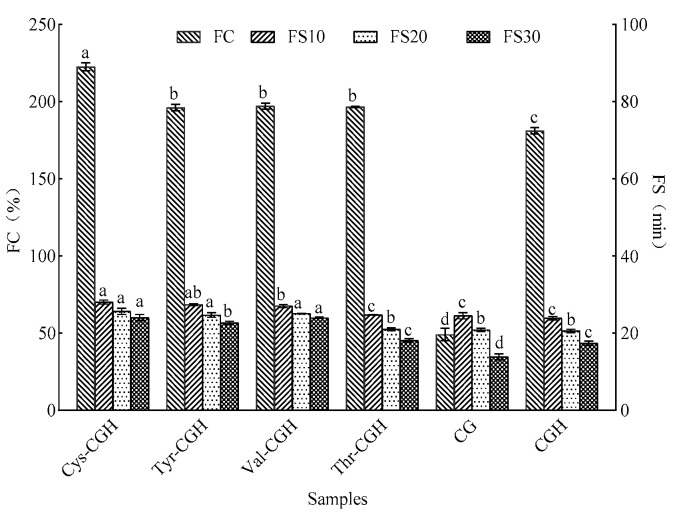
The changes in foamability and foaming stability in different samples. Note: A one-way ANOVA was conducted. Different lowercase letters display significant differences among groups (*p* < 0.05). Cys-CGH: cysteine modifier; Tyr-CGH: tyrosine modifier; Val-CGH: valine modifier; Thr-CGH: threonine modifier; CG: corn glutelin; CGH: corn glutelin hydrolysate.

The measurement results of the foamability and foam stability are displayed in Figure 3. Compared with CGH, the FC of Cys-CGH, Tyr-CGH, Val-CGH and Thr-CGH significantly increased by 41.5%, 15%, 16% and 15.5% respectively (*p* < 0.05). The foaming stability (FS10) of Cys-CGH (70.11%), Tyr-CGH (68.37%) and Val-CGH (67.51%) was significantly higher than that of CGH (59.67%) at 10 min (*p* < 0.05). Cys-CGH (64.04%), Tyr-CGH (61.73%) and Val-CGH (62.69%) still exhibited relatively stable foaming at 20 min (*p* < 0.05), compared with CGH (51.38%). The foaming stability (FS30) of Cys-CGH (60.00%), Tyr-CGH (56.63%) and Val-CGH (59.90%) was significantly higher than that of CGH (43.37%) at 30 min (*p* < 0.05). Meanwhile, it is evident that the foaming property and foaming stability of Cys-CGH surpass those of Tyr-CGH, Val-CGH and Thr-CGH. Therefore, due to its good performance in foaming ability and foam stability, Cys-CGH was used for subsequent bread experiments. The foaming properties of proteins are influenced by various factors, such as protein concentration, surface activity, secondary structure, molecular rigidity and flexibility, hydrophobicity and hydrophilicity, disulfide bonds and so on [43]. However, the plastein reaction increases the exposure of hydrophobic groups, and these changes promote the formation and stability of the foam network structure [14]. Simultaneously, the introduction of valine enhances the hydrophobicity of the peptide chain. The results show that hydrophobic amino acids exhibit strong adsorption ability at the gas–liquid interface, which helps to reduce the surface tension and promote the formation of foam [44]. Threonine is a polar amino acid with hydroxyl side chains, which can form multiple hydrogen bonds with water molecules, enhancing its dispersibility in the aqueous phase, promoting the rapid diffusion of proteins to the interface, and reducing the surface tension. This efficient interfacial adsorption behavior can enhance the initial foam generation capability [45]. Tyrosine, due to its benzene ring structure, displays strong hydrophobicity at the gas–liquid interface, enabling it to adsorb more effectively and reduce surface tension, thus promoting the formation of foam. Previous studies have shown that the surface hydrophobicity of proteins or peptides is positively correlated with their foaming ability [46]. Cysteine, as a sulfur-containing amino acid, possesses special potential in regulating protein structure and interfacial behavior due to its unique chemical activity of the sulfhydryl (-SH) group. The loosening of the structure mediated by sulfhydryl improves the molecular fluidity, while the newly formed disulfide bonds provide sufficient crosslinking support, thus maintaining the structural integrity of foams. In the research on brewer’s grain protein (BSGP), ultrasonic treatment combined with glycosylation reaction could increase its surface hydrophobicity and exposure of sulfhydryl and improve its foaming performance [47].

**Figure 4:**
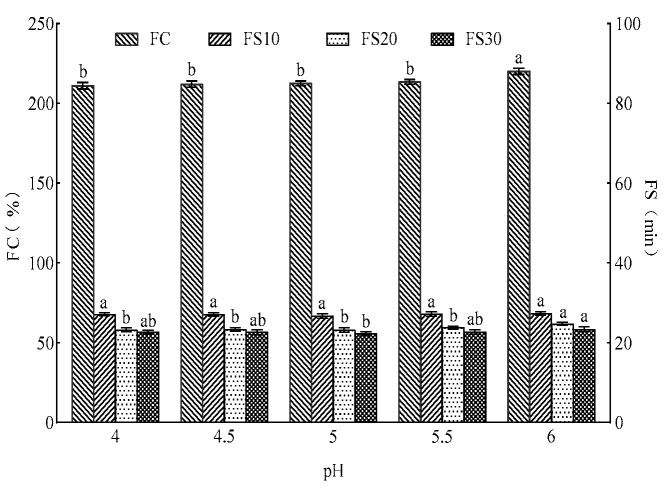
Changes in foaming capacity and foaming stability of Cys-CGH under different PH conditions. Note: A One-way ANOVA was implemented. Different letters display significant differences among groups (*p* < 0.05). Cys-CGH: cysteine modifier.

**Figure 5:**
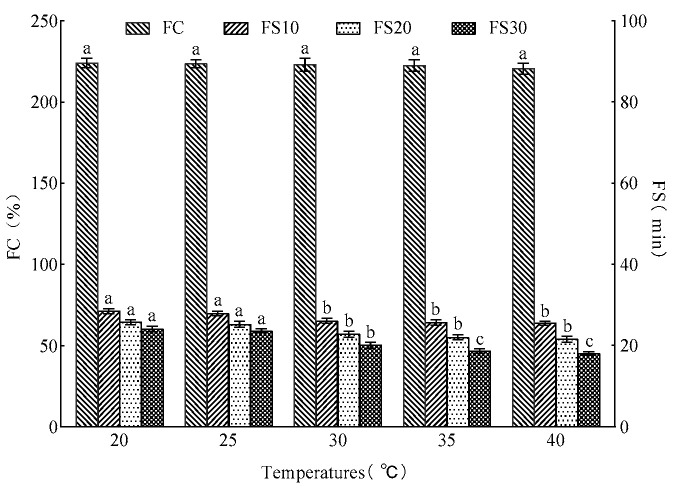
Changes in foaming capacity and foaming stability of Cys-CGH under different temperature conditions. Note: One-way ANOVA was carried out. Different letters suggest significant differences among groups (*p* < 0.05). Cys-CGH: cysteine modifier.

Figure 4 and Figure 5 show the influences of varying pH levels and different temperatures on the foaming capacity and foaming stability of Cys-CGH. Within the fermentation pH range of 4–6, Cys-CGH exhibits good foaming properties. The foaming capacity of CGH-Cys at pH 6 (222.0%) was significantly higher than that at pH 4 (211.0%), pH 4.5 (212.0%), pH 5 (212.5%) and pH 5.5 (213.5%) (*p* < 0.05), but it still showed strong foaming stability. The foamability of CGH-Cys was not significantly affected within the temperature range of 20–40 °C, but the foaming stability of CGH-Cys at 20 °C and 25 °C was significantly higher than that at 30 °C, 35 °C and 40 °C. Cys-CGH exhibits stronger foaming capacity at pH 6, attributed to its relatively high solubility, compared to that at pH 4–5.5. The high solubility of protein facilitates the diffusion from the aqueous phase to the interface and plays a positive role in the formation of bubbles [48]. As the temperature increases, the thermal movement of molecules intensifies, which weakens the cohesion between surfactant molecules, thereby reducing the mechanical strength of gas–liquid interface, subsequently decreasing the foam stability [49]. Within the temperature and PH range suitable for bread fermentation, Cys-CGH displays good foaming ability and foaming stability, which endows it with greater potential in the field of bakery products. In the bakery field, these modifiers can frequently be added as food-grade and high-quality function ingredients to be used as a substitute for chemical additives, which holds significant importance.

### 3.5. Particle Size and Zeta Potential Analysis of the Modified Products

**Figure 6:**
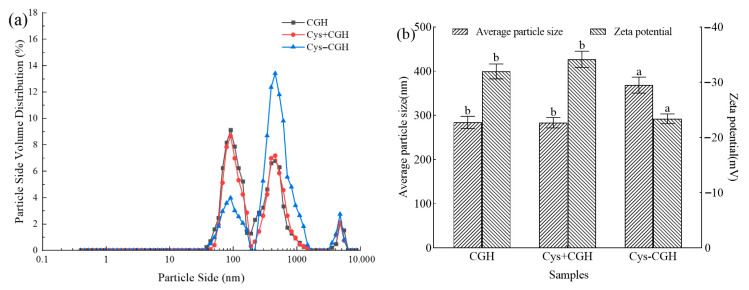
Changes in the particle size and Zeta potential of different modifiers; (**a**) Particle size volume distribution; (**b**) Average particle size and Zeta potential. Note: A one-way ANOVA was carried out in the analysis. Different letters display significant differences among groups (*p* < 0.05). CGH: Corn glutelin hydrolysate, Cys+CGH: physical mixture of cysteine and corn glutelin hydrolysate, Cys-CGH: a modifier for plastein reaction.

Particle size can reflect the stability of protein solution and reveal behaviors such as aggregation and depolymerization during protein hydrolysis processing [50]. The plastein reaction can promote the re-polymerization of small peptides, forming larger-scale aggregates. From Figure 6a, it can be seen that the particle size volume of CGH is mainly distributed around 100 nm, while the particle size volume of Cys-CGH modified by the plastein reaction is mainly distributed around 500 nm. The average Zeta potential and particle size of different samples are summarized in Figure 6b. The average particle size of Cys-CGH (368.63 nm) was significantly larger than that of CGH (284.21 nm) (*p* < 0.05). Compared with CGH, the average Zeta potential and particle size of Cys+CGH did not change significantly (*p* > 0.05). Compared with CGH, the absolute value of Zeta potential decreased by 8.58 mV (*p* < 0.05). Cysteine contains an active sulfhydryl group (-SH), which facilitates the formation of disulfide bonds (-S-S-) under oxidative conditions, providing an additional pathway for covalent linkage between peptide chains. This may further enhance the stability and size of aggregates. The elevation in absolute value of Zeta potential may be due to the decrease in the content of amino group (-NH_2_) after the plastein reaction modification. The study by Mohan et al. [16] showed that casein hydrolysate (CH) undergoes the plastein reaction to produce a modified product. The potential value of modifier (−22.3 mV) was significantly higher than that of CH (−33.6 mV), and the average particle size of modifier also increased accordingly. Cheng et al. reached a consistent conclusion when studying the protein hydrolysate of chicken lung treated by the plastein reaction, namely, the particle size of the modified product increased and the potential increased [14].

### 3.6. Scanning Electron Microscope Analysis of Different Samples

**Figure 7:**
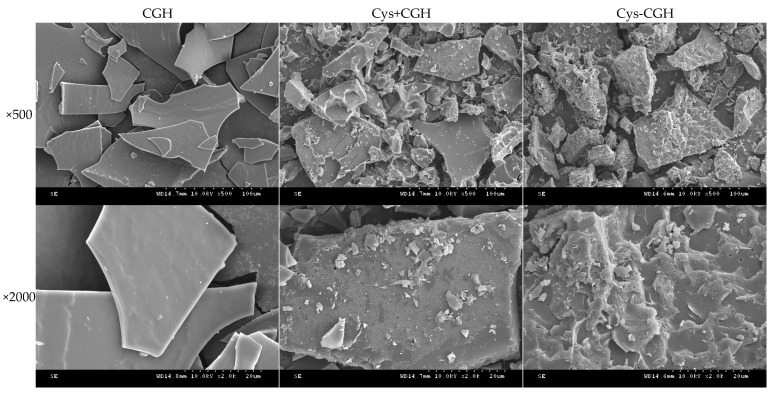
SEM images of different samples. Note: The magnification from top to bottom is ×500 and ×2000. The samples from left to right are: CGH: Corn glutelin hydrolysate, Cys+CGH: physical mixture of cysteine and corn glutelin hydrolysate, Cys-CGH: a modifier for plastein reaction.

Figure 7 reveals the microstructure changes in CGH before and after the plastein reaction, with significant differences observed among different treatment groups. The surface of CGH exhibited a dense lamellar structure with small pores. The surface of CGH+Cys, obtained by physically mixing CGH with Cys, showed a slight sheen and was coated with small patches of material, possibly due to the adhesion of Cys to the surface of the hydrolyzed product. The surface of CGH-Cys is not smooth but is covered with protuberant lines. Therefore, simple physical mixing did not significantly change the surface of the hydrolyzed product, which is further exemplified by the significant surface changes in the hydrolyzed product induced by the plastein reaction modification. The plastein reaction induces the repolymerization of peptide chains and the rearrangement of spatial conformation [51], thus changing the morphological characteristics of the sample surface. Cheng et al. [14] also verified that the surface of chicken lung protein hydrolysate (CLPH) by the plastein reaction modification (CLPH-P) was not smooth, but rather features angular protrusions, which support the results of this study.

### 3.7. Changes in Amino Acid Content and Composition of Different Samples

The amino acid compositions of the different samples are listed in Table 2. Glutamic acid had the highest content in CG, CGH and Cys-CGH. Xia et al. [52] obtained similar results in the study of barley glutenin, and the content of glutamic acid was also the highest in barley glutenin and hydrolysate. Compared with CG, the total level of hydrophobic amino acids in CGH decreased by 2.87 g/100 g protein. Compared with CG and CGH, the contents of sulfur-containing amino acids Cys and Met increased. During the plastein reaction process, the sulfhydryl group of cysteine can react with the oxidative groups or other active sites in the corn gluten hydrolysate. The exogenous cysteine is mediated into corn gluten hydrolysate through the plastein reaction, resulting in the increase in total sulfur-containing amino acids in the final product. The content of cysteine in Cys-CGH (7.75 g/100 g protein) was markedly higher than that in CG (0.30 g/100 g protein) and CGH (0.26 g/100 g protein). The results indicated that the plastein reaction introduced cysteine through covalent modification, altering the amino acid composition, which further explained the differences in physicochemical and functional properties between Cys-CGH and CGH.

### 3.8. Effect of the Addition of Modified Products on the Quality of Bread

#### 3.8.1. Effect of the Addition of Modifiers on Physical Characteristics of Bread

**Figure 8:**
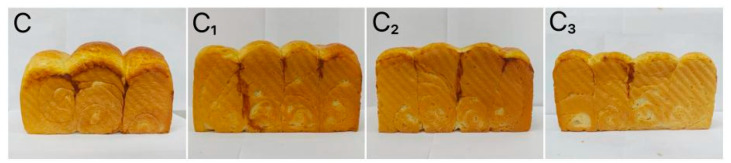
Changes in overall appearance of bread sample at different addition levels. Note: C: Control bread sample without adding Cys-CGH; C_1_: Bread sample containing 0.5% Cys-CGH; C_2_: Bread sample containing 1% Cys-CGH; C_3_: Bread sample containing 2% Cys-CGH.

Specific volume and baking loss rate are important indexes to evaluate the quality of baking products. The corresponding measurement results are presented in Table 3. The results suggested that the specific volume of the bread sample with 0.5% Cys-CGH (3.71 cm^3^/g) was higher than that of the control sample (3.44 cm^3^/g) (*p* < 0.05). Compared with the control sample, specific volume of the bread sample with 1.0% Cys-CGH increased slightly; however, the specific volume of the bread sample with 2% Cys-CGH decreased by 0.3 cm^3^/g. Compared to the control sample, the baking loss rate of the bread sample with 0.5% Cys-CGH, 1% Cys-CGH, and 2% Cys CGH reduced by 0.93%, 0.56%, and 0.12%, respectively. It can be inferred that the key reason for the rise in specific volume is the excellent foaming performance of Cys-CGH, which can enhance the stability of the bubble structure during baking. The covalent property of the plastein reaction stoichiometry leads to an increase in specific volume and decrease in baking loss. However, if the addition concentration of exogenous amino acids is too high, the continuity of the original gluten network may be destroyed due to excessive protein aggregation, resulting in a decrease in specific volume [53,54]. Figure 8 displays the influence of adding different amounts of Cys-CGH on the appearance of the bread sample. The bread sample with 0.5% Cys-CGH was significantly larger in volume, more compact, and stable in overall appearance than the other bread samples. This rise in volume brought about a diminution in hardness, while the bread sample exhibited a softer texture and lower density. From Table 4, it can be seen that compared to the control sample, the hardness of the bread sample containing 0.5% Cys-CGH was reduced by 0.3 N. This may be due to the appropriate addition of Cys-CGH, which enhances the mechanical strength and ductility of the gluten network, thereby improving its air-holding capacity. Zhang et al. [55] studied the effect of extracellular polysaccharides produced by lactic acid bacteria on the quality of buckwheat bread. The results indicated that compared with the control, the NC516.11 group increased the specific volume by 26.03% and decreased the baking loss by 19.94%. Zhang et al. [56] investigated the effect of wheat oligopeptides (WOP) on the baking quality of the bread roll. The addition of WOP (0.5%, 1% and 1.5%) can increase the specific volume of the bread roll. Adding 1% WOP led to the most significant enhancement in the specific volume, while adding 1.5% WOP decreased the specific volume.

**Figure 9:**
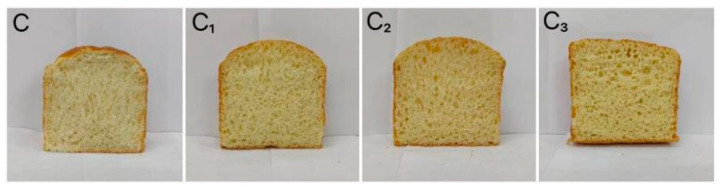
Changes in bread cross-section with different amounts of Cys-CGH addition. Note: C: Control bread sample without adding Cys-CGH; C_1_: Bread sample containing 0.5% Cys-CGH; C_2_: Bread sample containing 1% Cys-CGH; C_3_: Bread sample containing 2% Cys-CGH.

The assessment of porosity can penetrate the texture of baked goods. Higher porosity is generally associated with better specific volume, which endows bread with a light and soft taste, improving consumer acceptance. Figure 9 exhibits the effect of different addition levels on the bread cross-section. The bread sample with 0.5% Cys-CGH has more uniform and finer pores. The addition of a vegetation protein foam agent with a specific concentration can make the internal aperture of the bread more uniform and the distribution of the bread pores more concentrated [56]. Table 3 demonstrates the porosity of the bread samples, compared with the control sample, the porosity of the bread sample containing 0.5% Cys-CGH was clearly higher than that of control sample, increased by 2.55% (*p* < 0.05), the porosity of the bread sample with 2% Cys-CGH significantly decreased, decreased by 1.99% (*p* < 0.05), and the porosity of the bread sample including 1% Cys-CGH had no significant effect (*p* > 0.05). Cys-CGH can increase the formation of disulfide bonds in the molecule, thereby enhancing the elasticity and extensibility of gluten network. However, when the concentration of Cys-CGH is too high, it can damage the integrity of gluten network, leading to a decrease in porosity. Studies have suggested that higher porosity typically corresponds to a lighter, softer texture, while lower or unevenly distributed porosity may lead to products that are compact, dry and hard, with a decrease in overall sensory acceptance [57,58]. Atudorei et al. [59] confirmed that adding different levels of germinated lentil flour (LGF) (2.5%, 5%, 7.5% and 10%) to wheat flour used in bread making can increase the porosity of bread. When the amount of LGF increased from 2.5% to 7.5%, the porosity gradually increased and reached its highest at 7.5%. When 10% LGF was added, the porosity decreased, which was consistent with the results of this experiment.

#### 3.8.2. Influence of the Modifier Addition on Bread Color

The impact of adding modifiers on color of the bread sample crust and crumb is listed in Table 5. The color lightness (L) of bread crust with 0.5%, 1% and 2% Cys-CGH was evidently higher than that of the control (*p* < 0.05); the bread crumb color was a little higher than that of the control, but no obvious difference was observed (*p* > 0.05). Compared to the control sample, the red–green color constituent (a) of bread crust and crumb in the bread samples with 0.5%, 1% and 2% Cys-CGH obviously decreased (*p* < 0.05). For the yellow-blue color constituent (b), the bread crust color exhibited a slight reduction by adding 0.5%, 1% and 2% Cys-CGH, compared to the control, but no evident difference was observed (*p* > 0.05). The improvement in lightness (L) value of crust and crumb color may be due to Cys-CGH inhibiting browning. Amino acid compounds are one of the prominent factors affecting the Maillard reaction. The browning inhibition effect of cysteine can be attributed to its free thiol group with special redox and nucleophilic characteristics, which can easily react with carbonyl intermediates to suppress pigmentation [60]. Ho et al. [61] also proved that adding 10% BPF (bananapseudo-stem flour) to wheat bread can increase the lightness (L) of the bread crust by 5.11 and decrease the red–green components (a) by 2.52.

#### 3.8.3. Influences of the Modifier Addition on Texture Features of Bread Samples

Measurement results of the TPA of bread samples are displayed in Table 4. Compared against the control sample, the hardness of bread samples with 0.5% Cys-CGH visibly decreased by 0.3 N, the hardness of bread samples with 2% Cys-CGH evidently increased by 0.58 N (*p* < 0.05), and the hardness of bread samples with 1% Cys-CGH slightly decreased, but there was no evident difference (*p* > 0.05). Compared to the control, springiness of bread samples with 0.5% Cys-CGH and 1% Cys-CGH increased by 1.03 and 0.23, respectively, but the difference was not statistically evident (*p* > 0.05). Compared to the control, the chewiness of bread samples with 0.5% Cys-CGH and 1% Cys-CGH was significantly increased by 7.50 N and 3.68 N, respectively, and the chewiness of bread samples with 0.5% Cys-CGH was clearly higher than that of 1% Cys-CGH (*p* < 0.05). Compared with the control, and the Cohesion of bread samples with 0.5% Cys-CGH, 1% Cys-CGH and 2% Cys-CGH slightly decreased, but there was no clear difference (*p* > 0.05). In actuality, one study has found that adding soy protein isolate during bread making can observably reduce the hardness of bread [62]. Compared with the control, adding 4% soy protein isolate could reduce the hardness of bread by 1.19 N. Another research on the effect of adding whole quinoa flour on bread quality demonstrated that the springiness and chewiness of bread samples clearly improved [63]. Compared with the control, adding 30% whole quinoa flour could significantly increase the springiness and chewiness by 0.042 and 72.93 N, respectively. Moreover, research showed that adding 5%, 10% and 15% pea protein concentrate to wheat bread could remarkably increase the chewiness of wheat bread (*p* < 0.05) [64].

#### 3.8.4. Electronic Tongue Analysis of Bread Samples

**Figure 10:**
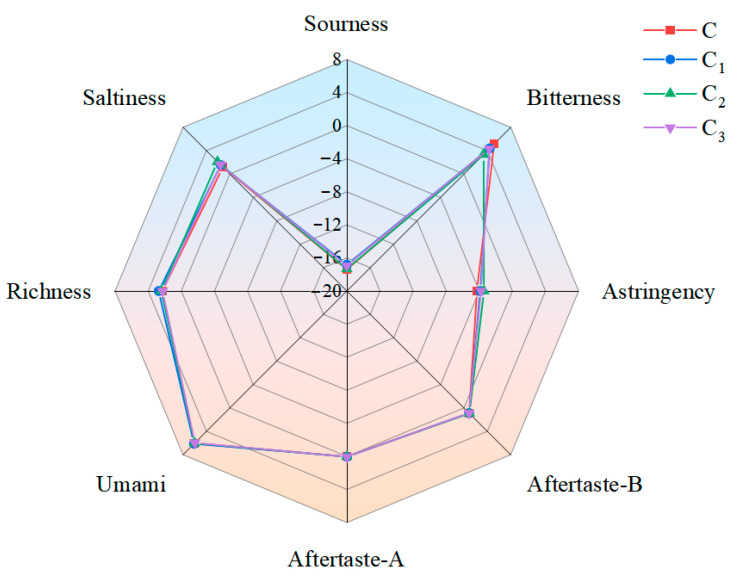
Electronic tongue measurement results of different bread samples. Note: red squares denote group C (control bread sample without Cys-CGH); C_1_: blue circles denote group C_1 _(Bread sample containing 0.5% Cys-CGH); C_2_: green triangles denote group C_2_ (bread sample containing 1% Cys-CGH; C_3_: purple inverted triangles denote group C_3_ (bread sample containing 2% Cys-CGH.

Electronic tongue detection may effectively ascertain subtle taste differences in food with no subjective bias, rendering it a mighty tool for differentiating taste features [65]. Figure 10 expresses the detection results of the electronic tongue for different bread samples. Compared to the control, the sourness of bread samples with Cys-CGH (0.5% and 2%) rose significantly by 0.57 and 0.46, respectively. Bitterness of bread samples added with Cys-CGH (0.5%, 1%, and 2%) decreased by 0.76, 1.78 and 1.83. The addition of Cys-CGH (1% and 2%) significantly increased the astringency of bread samples (*p* < 0.05); however, the addition of 0.5% Cys-CGH had no significant influence on the astringency of bread samples (*p* > 0.05). Aftertaste-B and aftertaste-A of bread samples were not significantly affected (*p* > 0.05). Compared with the control, the umami taste of bread samples with 0.5% Cys-CGH slightly increased, and the umami taste of bread samples with 1% Cys-CGH and 2% Cys CGH showed a modest increase. The flavor richness of the bread samples with 0.5% Cys-CGH was evidently higher than that of the control, the bread samples with Cys-CGH (1% and 2%) (*p* < 0.05). Adding Cys-CGH can reduce the bitterness of bread, which may be due to Cys’ ability to inhibit browning, slow down the Maillard reaction, and prevent the formation of macromolecular black dextrin during baking [60]. The increase in umami and richness may also be related to the Maillard reaction; meanwhile, Yan et al. [66] found that moderate Maillard reaction enhances the flavor characteristics of Maillard reaction products.

## 4. Conclusions

Corn glutelin hydrolysate (CGH) was prepared by hydrolyzing corn glutelin with alcalase. The plastein reaction mediated the modification of corn glutelin hydrolysate by exogenous amino acids (valine, tyrosine, cysteine, and threonine). The synthesized Val-CGH, Tyr-CGH, Cys-CGH and Thr-CGH have enhanced solubility, foamability and foam stability. In addition, the bread product containing 0.5% Cys-CGH displayed better quality, resulting in incremental specific volume, less baking loss, lower firmness, increased springiness, improved umami, and richness.

This study introduced exogenous amino acids through the plastein reaction to modify corn glutelin hydrolysates, revealing the synergistic control of the plastein reaction and different amino acids on its structure features and function properties. This provides a theoretical basis for evaluating the functional properties of corn glutelin. In addition, the researchers innovatively applied the modified products to bread formulations to explore their potential in improving the quality and functionality of bread. The results of this study have developed a new functional ingredient for the baking food industry and provided feasible solutions, which not only improve the functional characteristics and nutritional quality of protein, but also broaden the high-value utilization pathways of agricultural by-products, promoting the development trend of functional foods.

## Figures and Tables

**Table 1:** Free amino level of different samples.

Samples	Classification	–NH_2_ Level (mmol/g Protein)
CG	corn glutelin	0.0231 ± 0.0013 ^g^
CGH	corn glutelin hydrolysate	1.7928 ± 0.0170 ^c^
Val-CGH	The plastein modified product of CGH and Val	1.4358 ± 0.0210 ^f^
Val+CGH	The Physical mixture of CGH and Val	1.9136 ± 0.0150 ^b^
Thr-CGH	The plastein modified product of CGH and Thr	1.5282 ± 0.0100 ^e^
Thr+CGH	The physical mixture of CGH and Thr	1.9738 ± 0.0160 ^a^
Tyr-CGH	The plastein modified product of CGH and Tyr	1.6017 ± 0.0140 ^d^
Tyr+CGH	The mixture of CGH and Tyr	1.8903 ± 0.0110 ^b^
Cys-CGH	The plastein modified product of CGH and Cys	1.5458 ± 0.0190 ^e^
Cys+CGH	The mixture of CGH and Cys	1.9824 ± 0.0130 ^a^

Note: Experiment results are displayed as mean ± standard deviation (SD), n = 3. Different superscript lowercase letters (a–g) in the same column demonstrate significant differences among groups, and one-way analysis of variance (ANOVA) was employed (*p* < 0.05).

**Table 2:** Amino acid content of different samples.

Amino Acid	Amino Acid Content (g/100 g)		
	CG	CGH	Cys-CGH
Asp	4.35	3.98	3.77
Thr	2.61	2.31	2.21
Ser	3.32	2.98	2.83
Glu	12.60	11.80	11.20
Gly	2.79	2.49	2.35
Ala	5.60	5.13	4.50
Cys	0.30	0.26	7.75
Val	3.76	3.33	3.10
Met	1.88	2.21	2.44
Ile	2.85	2.65	2.45
Leu	9.81	8.94	8.18
Tyr	3.79	3.57	3.40
Phe	4.26	3.96	3.71
Lys	1.95	1.92	1.89
His	2.03	1.79	1.69
Arg	2.97	2.54	2.39
Pro	6.96	6.03	5.70

Note: CG: Corn glutelin, CGH: Corn glutelin hydrolysate, Cys-CGH: a modifier for plastein reaction.

**Table 3:** Changes in physical characteristics of the bread sample after the modifier addition.

Samples	Specific Volume (cm^3^/g)	Baking Loss (%)	Porosity (%)
C	3.44 ± 0.10 ^b^	7.82 ± 0.29 ^a^	29.29 ± 0.80 ^b^
C_1_	3.71 ± 0.12 ^a^	6.89 ± 0.32 ^b^	31.84 ± 1.00 ^a^
C_2_	3.58 ± 0.11 ^ab^	7.26 ± 0.24 ^ab^	29.38 ± 0.73 ^b^
C_3_	3.14 ± 0.08 ^c^	7.70 ± 0.51 ^ab^	27.30 ± 0.78 ^c^

Note: Experimental results are shown as mean ± standard deviation (SD), n = 3. Data statistics confirmed by a one-way analysis of variance (ANOVA), and superscripts of different lowercase letters (a–c) derived from same column demonstrate obvious differences among groups (*p* < 0.05). C: bread sample without Cys-CGH addition; C_1_: Bread sample containing 0.5% Cys-CGH; C_2_: Bread sample containing 1% Cys-CGH; C_3_: Bread sample containing 2% Cys-CGH.

**Table 4:** Changes in texture characteristics of bread sample after the modifier addition.

Samples	Hardness (N)	Springiness (mm)	Chewiness (N)	Cohesion (1)
C	5.51 ± 0.39 ^ab^	10.49 ± 0.43 ^ab^	38.55 ± 1.54 ^c^	0.63 ± 0.03 ^a^
C_1_	5.21 ± 0.13 ^b^	11.52 ± 0.62 ^a^	46.05 ± 1.42 ^a^	0.61 ± 0.01 ^a^
C_2_	5.48 ± 0.18 ^ab^	10.72 ± 0.27 ^ab^	42.23 ± 1.04 ^b^	0.61 ± 0.03 ^a^
C_3_	6.09 ± 0.36 ^a^	10.12 ± 0.74 ^b^	37.17 ± 1.33 ^c^	0.60 ± 0.02 ^a^

Note: Experiment results are shown as mean ± standard deviation, n = 3. Using a one-way analysis of variance, different lowercase letters (a–c) above the same column exhibit obvious differences among groups (*p* < 0.05). C: Control bread sample without adding Cys-CGH; C_1_: Bread sample including 0.5% Cys-CGH; C_2_: Bread sample including 1% Cys-CGH; C_3_: Bread sample including 2% Cys-CGH.

**Table 5:** Changes in the color of the bread sample after the modifier addition.

Samples		C	C_1_	C_2_	C_3_
Crust color	L	39.79 ± 0.51 ^c^	44.51 ± 0.91 ^a^	41.56 ± 0.56 ^c^	41.58 ± 0.50 ^b^
	a	17.09 ± 0.82 ^a^	10.99 ± 0.47 ^b^	11.05 ± 0.91 ^b^	10.12 ± 0.58 ^b^
	b	24.14 ± 0.78 ^a^	22.61 ± 0.81 ^a^	23.03 ± 1.26 ^a^	22.71 ± 0.69 ^a^
Crumb color	L	25.52 ± 0.64 ^a^	25.75 ± 0.05 ^a^	26.56 ± 0.87 ^a^	27.53 ± 0.96 ^a^
	a	2.13 ± 0.58 ^a^	0.69 ± 0.23 ^b^	081 ± 0.36 ^b^	1.03 ± 0.48 ^b^
	b	12.96 ± 0.62 ^ab^	12.86 ± 0.54 ^ab^	13.75 ± 0.17 ^a^	12.41 ± 0.16 ^c^

Note: The experimental data are displayed as mean ± standard deviation (SD), n = 3. All data are carried out using a one-way analysis of variance. Superscripts in different lowercase letters (a–c) derived from the same column demonstrate clear differences among groups (*p* < 0.05). C: Bread sample without Cys-CGH; C_1_: Bread sample containing 0.5% Cys-CGH; C_2_: Bread sample containing 1% Cys-CGH; C_3_: Bread sample containing 2% Cys-CGH.

## Data Availability

The original contributions presented in this study are included in the article. Further inquiries can be directed to the corresponding authors.
